# Application of Fourier-Transform Infrared Spectroscopy for the Assessment of Wine Spoilage Indicators: A Feasibility Study

**DOI:** 10.3390/molecules29081882

**Published:** 2024-04-20

**Authors:** Cláudia Andreia Teixeira dos Santos, Ricardo Nuno Mendes Jorge Páscoa, Nuria Pérez-del-Notario, José Maria González-Sáiz, Consuelo Pizarro, João Almeida Lopes

**Affiliations:** 1 REQUIMTE, Laboratório de Química Aplicada, Departamento de Ciências Químicas, Faculdade de Farmácia, Universidade do Porto, Rua de Jorge Viterbo Ferreira, 228, 4050-313 Porto, Portugal; 2 Departmento de Química, Universidad de La Rioja, C/Madre de Dios 51, 26006 Logrono, La Rioja, Spain; 3 Research Institute for Medicines (iMed.ULisboa), Faculdade de Farmácia, Universidade de Lisboa, Av. Prof. Gama Pinto, 1649-003 Lisbon, Portugal

**Keywords:** Fourier-transform infrared spectroscopy, wine off-odors, odor thresholds, early detection, novel technique, chemometrics

## Abstract

Wine aroma is one of the most frequently used and explored quality indicators. Typically, its assessment involves estimating the volatile composition of wine or highly trained assessors conducting sensory analysis. However, current methodologies rely on slow, expensive and complicated analytical procedures. Additionally, sensory evaluation is inherently subjective in nature. Therefore, the aim of this work is to verify the feasibility of using FTIR spectroscopy as a fast and easy methodology for the early detection of some of the most common off-odors in wines. FTIR spectroscopy was combined with partial least squares (PLS) regression for the simultaneous measurement of isoamyl alcohol, isobutanol, 1-hexanol, butyric acid, isobutyric acid, decanoic acid, ethyl acetate, furfural and acetoin. The precision and accuracy of developed calibration models (R^2^_P_ > 0.90, range error ratio > 12.1 and RPD > 3.1) proved the ability of the proposed methodology to quantify the aforementioned compounds.

## 1. Introduction

Wine is one of the most complex alcoholic beverages. Several hundreds of chemical compounds define its character and quality. Chemical and organoleptic analyses are typically employed to assess wine quality, with wine aroma emerging as the most widely used and explored quality indicator. In fact, wine aroma arises from a complex blend of volatile molecules originating from diverse and interrelated sources: grape variety and associated *terroir*, microbiological pathways followed during alcoholic and malolactic fermentation, vinification process and aging/storage conditions [1,2,3]. Therefore, in addition to assessing wine quality, analyzing the volatile composition of wine can provide valuable insights for wine characterization and the optimization of viticultural and winemaking practices.

In recent years, several studies have reported an extensive number of volatile compounds present in wine aroma, including alcohols, acids, esters, phenols, aldehydes, thiols, monoterpenes and ketones, among others [1,4]. Their contribution to wine aroma is determined by their concentration and perception threshold, with only a subset considered as impact odorants [1,4,5].

At high concentrations, compounds such as higher alcohols, volatile fatty acids, ethyl acetate, acetoin and furfural are related to unpleasant sensorial characteristics in wines. These odorants, commonly assigned as off-odors, are typically produced by yeasts or bacteria in response to unfavorable conditions during alcoholic and malolactic fermentation. Thus, it is crucial to measure and control these aromas before they exceed their odor thresholds in wine.

Higher alcohols are often associated with the fruity character of wines. However, at high concentrations, isoamyl and isobutyl alcohols introduce strong and pungent odors, while 1-hexanol imparts an herbaceous scent that can overlap the overall aroma of wine. Volatile fatty acids, namely butyric, isobutyric and decanoic acids, when present above their odor threshold, contribute to fatty, cheesy and sweaty aromas in wine. They may also exhibit fermentation-inhibitory effects to some extent. Ethyl acetate, typically considered the most significant ester in wines, is primarily responsible for its off-odor, often described as glue- or acetone-like. Acetoin, among the ketones, is recognized for its flavor significance in wine. Its presence at high concentrations adds a buttery aroma to the overall bouquet. Furfural, an aldehyde found in wine, imparts toasty, almond and caramel-like tones, contributing to the baked character of wines [3,6,7,8,9].

Thus, it is imperative to develop an analytical method capable of determining all these compounds, either individually or simultaneously, in wines. The reference procedures involve extraction techniques, followed by slow, expensive and complicated analytical procedures (such as gas chromatography) [10]. Additionally, sensory analysis, also employed in these situations, relies on a highly trained panel of sensory assessors, which is time-consuming and expensive. In the worst-case scenario, these compounds are only detected when their presence in wine already constitutes a defect. More recently, the application of two-dimensional (2D) gas chromatography for the characterization of volatile compounds has been proposed as a promising alternative due to its higher capacity to detect a wider range of volatile compounds and at lower concentrations [11]. However, it still requires time-consuming sample preparation steps.

Fourier-transform infrared (FTIR) spectroscopy offers a fast, non-destructive and cost-effective technique with a high level of precision, accuracy and reproducibility. In fact, this technique has been widely used in the wine industry for various purposes, including the identification and/or quantification of compounds [12,13,14,15], fermentation monitoring [16], authentication of products and fraud detection [17].

Chemometric techniques, preprocessing tools and calibration procedures, when properly combined, provide the necessary support to overcome some of the limitations associated with FTIR spectroscopy. Water and ethanol, as the major components of wine, introduce dominant bands in the spectrum, thereby disrupting the measurement of minor compounds. The high degree of chemical similarity among wine constituents further complicates the measurement process. Finally, the relatively low sensibility of FTIR instruments represents one of the main drawbacks associated with this technique. However, the significant advantages of FTIR spectroscopy are worth the effort to develop and expand its use to several other applications.

Therefore, the aim of this work is to test the feasibility of FTIR spectroscopy for measuring some of the most common off-odors (as the ones listed in Table 1), typically associated with wine defects. The lower sensitivity of the FTIR technique triggered the selection of compounds with higher odor threshold values. Consequently, isoamyl alcohol, isobutanol, 1-hexanol, butyric acid, isobutyric acid, decanoic acid, ethyl acetate, acetoin and furfural were chosen for investigation. To the best of our knowledge, this is the first time such methods have been developed for the quantitative determination of these compounds in wine using FTIR spectroscopy.

## 2. Results and Discussion

### 2.1. Calibration Procedure and Statistics

As explained earlier, spectra were divided into five spectral regions assigned as region 1 (1200 to 950 cm^−1^), region 2 (1580 to 1201 cm^−1^), region 3 (1800 to 1581 cm^−1^), region 4 (2600 to 1801 cm^−1^) and region 5 (3050 to 2601 cm^−1^). All these five regions underwent preprocessing and were submitted to PLS regression, either individually or in combination, resulting in several calibration models for each compound. Although there are several variable selection methods available in the literature such as successive projections algorithm (SPA), uninformative variable elimination (UVE), and interval selection methods such as interval partial least squares (iPLS), among others (for more information, please refer to the following reference) [21], we decided to use the manual approach due to previous knowledge from our research group. In this context, spectral region 4 was found to lack valuable information, spectral region 3 was suspected to contain chemical information but was not useful for these PLS models, and spectral regions 1 and 2 were divided because they contained valuable but distinct chemical information. The resulting models were evaluated in terms of the coefficient of determination (R^2^) and associated predictive errors (RMSEs), enabling the identification of the best regions and preprocessing techniques for each specific compound. Regions 2 and 5 appeared to contain the most contributive and valuable information for the calibration of the nine parameters proposed here. The Savitzky–Golay method with the first derivative was found to be the most useful preprocessing technique. After model optimization, the test sets were projected. The statistical results for all PLS calibration models are listed in Table 2. Results considering the full spectrum (regions 1, 2, 3, 4 and 5 altogether) are presented in the Supplementary Materials (Table S1). It is evident that the selection of the best spectral regions leads to a significant improvement in the PLS results for all the models, as expected.

The criterion defined by Shenk and Westerhaus (1996) proposes that an R^2^ greater than 0.90 indicates “excellent” regression models capable of quantitative determinations [22]. According to this criterion, all developed models (especially the ones for ethyl acetate (R^2^_P_ = 0.98), furfural (R^2^_P_ = 0.97), acetoin (R^2^_P_ = 0.96) and 1-hexanol (R^2^_P_ = 0.96)) are remarkably capable of quantitative predictions. It is also worth noting that all predictive errors represent less than 10% of their respective concentration ranges. Regarding the RER parameter, when RER is higher than 4, the model can be used for screening; when it exceeds 10, the model can be employed for quality control; and when higher than 15, the model is considered very good for research quantification [23]. As for the RPD parameter, when its value ranges from 2.0 to 2.5, the model can be used for approximate quantifications; between 2.5 and 3.0, the developed models can be classified as good; and when exceeding 3.0, the developed models can be considered excellent [24].

**Figure 1:**
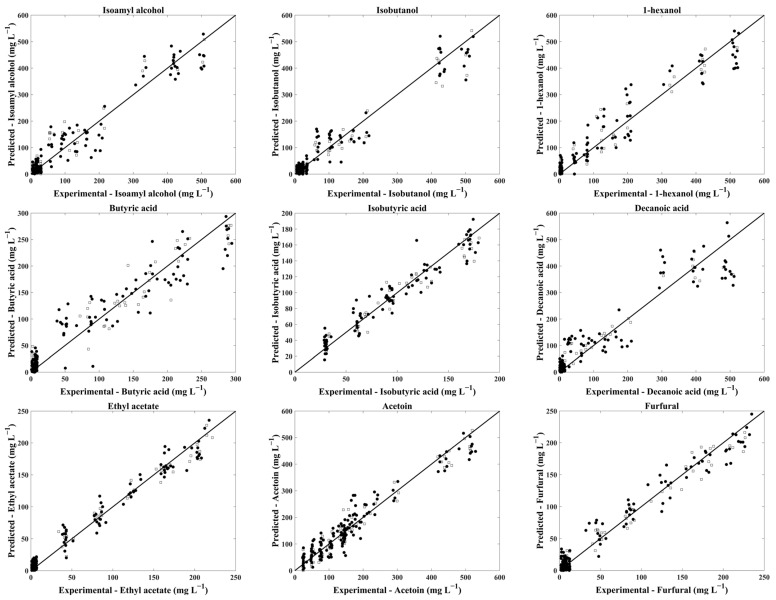
PLS regression models for cross-validation (●) and test sets (□) for isoamyl alcohol, isobutanol, 1-hexanol, butyric acid, isobutyric acid, decanoic acid, ethyl acetate, furfural and acetoin.

A comparison between experimental values of compound concentrations and corresponding PLS models’ predictions is given in Figure 1.

### 2.2. Models’ Interpretation

**Figure 2:**
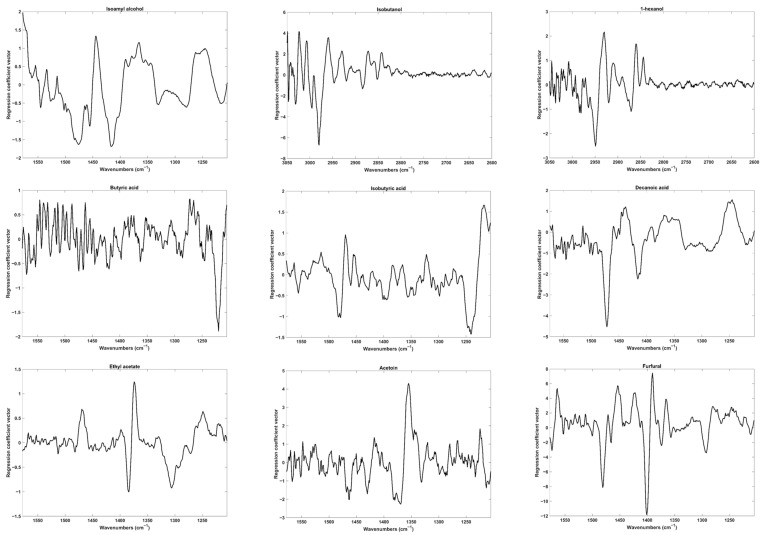
Regression coefficients’ vectors for all PLS-1 models.

The mid-infrared spectrum of wine is primarily dominated by intense water and ethanol absorption bands. These absorption bands pose a major limitation in determining higher alcohols. Additionally, the chemical similarity between wine constituents results in similar absorption features, further complicating the determination of minor compounds. The overlapped absorptions, typical in the FTIR spectra of wine, hinder direct interpretation and the observed peaks or bands cannot be assigned to individual compounds. Therefore, the plot of the regression coefficient vectors, as shown in Figure 2, serves as an alternative to support the selection of the most contributive regions for each compound.

The calibration of higher alcohols was conducted in two distinct spectral regions. Isobutanol and 1-hexanol were calibrated in region 5 (3050–2601 cm^−1^), whereas isoamyl alcohol yielded better results in region 2 (1580–1201 cm^−1^). The regression coefficients suggest that spectroscopic variations in region 5 are probably due to the CH_2_ symmetric and asymmetric stretching (reported at 2935–2840 cm^−1^ and 2990–2900 cm^−1^, respectively) [25], while for isoamyl alcohol, variations in region 2 may be correlated with –CH_2_, C–C–H and H–C–O deformation vibrations [26]. Moreover, region 2 also appeared to contain valuable information regarding the calibration of volatile fatty acids (Figure 2). Indeed, the C–O stretching and the O–H bending, as reported by Regmi (2012) around 1321–1210 cm^−1^ and 1420–1320 cm^−1^, respectively, seemed to contribute significantly to the successful calibrations of butyric acid, isobutyric acid and decanoic acid [27].

The best calibration models for furfural, acetoin and ethyl acetate were also obtained when region 2 was considered. For the ester ethyl acetate, the main contributions are observed around 1200–1350 cm^−1^ and 1400–1365 cm^−1^. In fact, the CH_3_ symmetric and asymmetric deformations, as well as the CO–O stretching, have been reported for acetates near 1375 cm^−1^, 1430 cm^−1^ and 1265–1205 cm^−1^, respectively. The presence of carbonyl and hydroxyl groups in acetoin makes it very similar to the major constituents of wine. However, spectral variations around 1400–1330 cm^−1^ were sufficient to produce a calibration model with an RER and RPD of 16.4 and 4.0, respectively. These variations may be associated with the C–C stretching vibration reported at 1325–1115 cm^−1^ for aliphatic ketones [21]. Furfural, a furan derivative aldehyde, exhibits high spectroscopic variations around 1500–1350 cm^−1^. These variations may be associated either with the C=C stretching in the furan ring (1400–1390 cm^−1^) or with the in-plane C–H rocking vibration of the aldehyde functional group (1450–1325 cm^−1^) [25].

### 2.3. Methods’ Evaluation

It was our priority to obtain PLS models with an LOD lower than the odor threshold value for each compound. This objective was particularly successful for isobutyric acid (LOD = 36.9 mg/L, RMSEP = 12.30 mg/L) and acetoin (LOD = 92.7 mg/L, RMSEP = 30.9 mg/L), as their LODs and predictive errors are lower than the respective odor threshold values. It is worth noting that the calibration model developed for isobutyric acid covers a concentration range (29.59–179.91 mg/L) below its odor threshold value (200 mg/L).

For all other six compounds, the limits of detection are higher than the respective odor threshold values. However, it is important to consider that these sensorial detection limits were established using model solutions. Assessing the perception of these compounds in wines would be more challenging due to the complexity of their matrix. Consequently, the odor threshold values in wines are typically much higher than those obtained for model solutions. From this perspective, the obtained LOD values may be considered suitable for the wine industry purposes.

It is true that reference procedures, such as gas chromatography and 2D gas chromatography, offer a lower LOD than the developed methodology herein, and are capable of accurately detecting and determining most of the chemical volatile compounds present in wines. In fact, these techniques are crucial for determining several volatile compounds present at very low concentrations and for developing PLS models. However, the methodology developed in this study, when compared to the laborious, time-consuming, environmentally harmful gas chromatographic techniques, offers several advantages. It is simpler, more rapid, cost-effective and aligned with Green Chemistry principles, as it does not require the use of reagents or produce effluents. In comparison to sensorial analysis, this method is also more rapid, cost-effective and does not depend on highly trained assessors.

Overall, nine PLS-based calibration models were proposed for the determination of off-odors in wines from FTIR spectra. The results revealed excellent regression properties (R_P_^2^ > 0.90, RER > 10.1 and RPD > 3.1) for isoamyl alcohol, isobutanol, 1-hexanol, butyric acid, isobutyric acid, decanoic acid, ethyl acetate, acetoin and furfural. The LOD values obtained for isobutyric acid and acetoin enhance the ability of this technique for the early detection of these compounds, with acceptably low errors of prediction before they become wine defects. Special attention should be given to isobutyric acid, whose calibration was performed in a range of concentrations below its odor threshold value. For the remaining seven compounds, the suitability of this technique was compromised by high LODs versus low odor threshold limits. However, as already mentioned, the complexity of the wine matrix makes the sensory perception of these compounds more difficult, thereby increasing their odor threshold values. To attest to the robustness of the proposed models, further studies should be performed, including a wide number and diversity of wine samples, and establishing the sensory thresholds of these compounds for those samples.

## 3. Materials and Methods

### 3.1. Samples’ Preparation

A young red wine from the Spanish DO La Rioja was selected for this study. The wine was kindly donated by Bodegas Riojanas winery located in the north of Spain, where the entire vinification process took place. The sample was retrieved directly from the cellar and stored at 14 °C until analysis. The sample exhibited the following parameters: pH of 3.6, ethanol concentration of 13.8%, total SO_2_ amount of 60 mg/L, total acidity of 5.2 g/L and residual sugars below 2 g/L.

The aim of this study was to assess the suitability of FTIR spectroscopy to determine the compounds under investigation: isoamyl alcohol, isobutanol, 1-hexanol, butyric acid, isobutyric acid, decanoic acid, ethyl acetate, acetoin and furfural in wine. As a first approach, a single matrix was considered to minimize potential interferences related to the wine’s compositional matrix. If our goal was to validate this method, additional samples encompassing a diverse range of wines (including different grapevine varieties, fermentation procedures and geographic origins, among others) would be necessary. Sensory analysis of this wine sample was conducted at Bodegas Riojanas winery by an expert winemaker and a group of trained experts belonging to the Wine Tasting Committee. No organoleptic faults were detected.

For the proper development of calibration models, each parameter’s concentration was increased by subjecting the wine to controlled additions (spiking). Before the additions (spiking) and subsequent FTIR measurements, the wine was centrifuged at 11,000 rotations per minute for 10 min at a temperature of 10 °C (Eppendorf centrifuge 5403—NY, USA) to ensure the homogenization of the sample. Standard solutions of isoamyl alcohol, isobutanol, 1-hexanol, butyric acid, isobutyric acid, decanoic acid and furfural (all supplied by Sigma-Aldrich, St. Louis, MO, USA), ethyl acetate (supplied by Scharlau, Barcelona, Spain) and acetoin (supplied by Fluka, Buchs, Switzerland) were prepared in methanol (supplied by Sigma-Aldrich). The high concentrations of these solutions ensured minimal additions, thereby avoiding significant changes in the original matrix. Previous calculations ensured that added volumes always represented less than 5% of the total sample volume. To ensure the reliability of this procedure, every single volume of standard solution added to the sample was weighed.

The concentration intervals selected for each compound’s calibration were determined based on their odor threshold values and the concentration of the solution used to prepare the standard solutions. Sensory thresholds are commonly determined by adding known concentrations of the pure compound to a model solution, usually comprising mixtures of water, ethanol, glycerin and tartaric acid. Trained panelists then evaluate the resulting solutions using the triangle test. Odor threshold values established by Guth (1997) or Ferreira (2000) are provided in Table 3 for each compound [28,29].

Considering the relatively low sensitivity of FTIR instruments, concentration limits were established between one-fifth of the odor threshold value and 200 mg/L for each compound. A total of 125 assays were conducted.

However, the initial calibration attempt using the selected concentration intervals revealed the unsuitability of the range for eight out of nine compounds. With the exception of isobutyric acid, it was necessary to expand the concentration intervals for all other compounds. Therefore, new experimental designs were conducted and the upper concentration levels were adjusted to 250 mg/L (for ethyl acetate and furfural), 300 mg/L (for butyric acid) and 500 mg/L (for isoamyl alcohol, isobutanol, 1-hexanol, decanoic acid and acetoin). This adjustment resulted in the addition of 98 new measurements or samples for each compound. The concentration ranges used for each parameter calibration, along with the respective odor threshold and the number of samples, are detailed in Table 3.

### 3.2. FTIR Analyses

FTIR spectral acquisition was performed using an ABB MB3000 (Québec, QC, Canada) spectrometer, equipped with Horizon MB (version 3.2.5.2) software. Measurements were the result of 32 scans, carried out in absorbance mode from 4000 to 300 cm^−1^, with a spectral resolution of 2 cm^−1^ (about 1 min of acquisition time). Sampling was conducted through a continuous-flow system, in which a peristaltic pump (Gilson Minipuls—Sarcelles, France) continuously pushed the sample to a CaF_2_ cell with an optical pathlength of 0.025 mm. Ten milliliters of sample, kept in a temperature-controlled room (25 °C), ensured triple measurement and preflushing of the system. Background measurements were taken against air before every set of 10 measurements.

### 3.3. Data Processing

**Figure 3:**
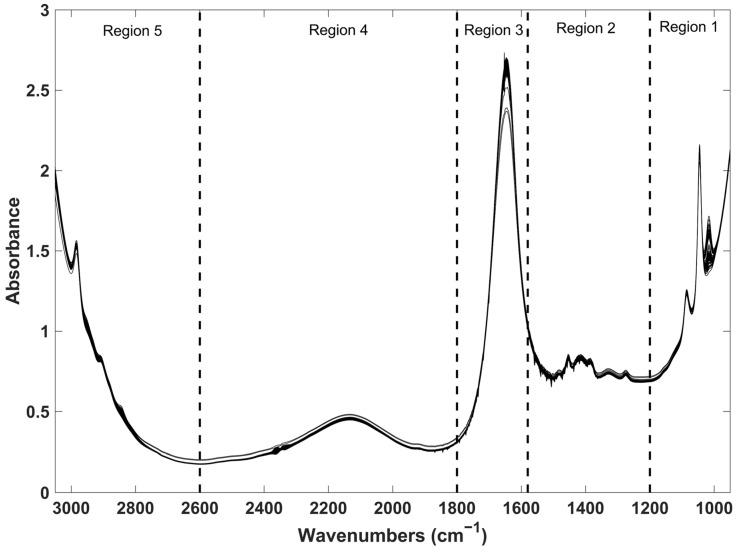
FTIR raw spectra of all wine samples used in this work.

A detailed exploratory analysis is frequently required when working with FTIR spectra to locate spectral regions encompassing the variation capable of providing useful results for each specific demand. A simple visual analysis of FTIR spectra led to the exclusion of the regions below 3050 cm^−1^ and above 950 cm^−1^. The remaining spectral section was subdivided into five spectral regions in agreement with the disposition of its peaks and/or bands (Figure 3). The five regions individually, as well as all their possible combinations (a total of 31 possibilities), were considered in the development of each compound’s calibration with the objective of ensuring an exhaustive investigation of the whole spectral range. Undesirable spectral variations generated by temperature variations, light scattering effects and baseline drifts may limit the proper use of the spectral data. Therefore, preprocessing techniques capable of reducing these undesirable effects were employed. Raw spectra were treated with standard normal variate (SNV) and/or Savitzky–Golay (with 15 points of filter width, second polynomial order) first- and second-order derivatives (individually or combined). SNV is commonly applied to remove scatter effects and to improve the signal-to-noise ratio by subtracting the spectra at each point from the average spectrum and then dividing by the respective standard deviation [29]. The Savitzy–Golay method is commonly applied to reduce the noise, improve the signal-to-noise ratio and correct for baseline variations. It basically finds the derivative of the spectrum via a smooth process, according to the window size selected (known as points of filter width) and polynomial degree order [30].

### 3.4. Multivariate Data Analysis

The following nine compounds in wine were considered: isoamyl alcohol, isobutanol, 1-hexanol, butyric acid, isobutyric acid, decanoic acid, ethyl acetate, furfural and acetoin. Before using the spectra, it was necessary to ensure their validity. To this purpose, a principal component analysis (PCA) model was created to identify possible outliers. This model was developed for the spectral regions between 1580 and 1201 cm^−1^ and between 3050 and 2601 cm^−1^, considering Savitzky–Golay first-order derivative spectra. The resulting PCA model encompassed three principal components that accounted for 95.6% of the total variance in the considered regions. From the analysis of the PCA model residuals (sum of squared residuals) and Hotelling’s T^2^ (weighted sum of squared scores) statistics, two samples were considered outliers. These samples were excluded from the sample sets.

Calibration models were built based on partial least squares (PLS) regression using the PLS-1 algorithm [31], by regressing processed spectral data against the corresponding concentration values obtained through controlled additions.

After developing the calibration models for each compound, it was crucial to evaluate the models and determine their predictive ability. One of the most commonly used procedures is based on the root mean square error of calibration (RMSEC) (Equation (1)).

(1) $$RMSEC=\sqrt{\frac{\sum_{i=1}^{N}{\left({\hat{y}}_{i}-y_{i}\right)}^{2}}{N}}$$

In Equation (1), *y_i_* represents the experimental measurement result for sample *i*, *ŷ_i_* denotes the model prediction for that sample and *N* stands for the number of samples used for calibration [32].

The calibration models underwent cross-validation using the leave-one-out technique, and subsequently, their performance was assessed using the root mean square error of cross-validation (RMSECV) according to Equation (1), where *ŷ_i_* represents the value predicted by the cross-validated model for sample i. This step also facilitated the selection of the optimal number of PLS factors, aiming for the lowest RMSECV value [33]. This process involved utilizing only 70% of the global dataset. The calibration models were also evaluated according to the coefficient of determination for calibration (R^2^_C_) and cross-validation (R^2^_CV_). Subsequently, the developed models were tested with independent datasets, representing the remaining 30% of the global dataset selected randomly. After projecting the test sets onto the models, their performance was evaluated again by calculating the RMSEP (Root Mean Square Error of Prediction). This error was calculated with *N* representing the number of samples in the prediction set and *ŷ_i_* representing the value obtained by the model for each sample i within the same sample set.

To express the previously defined errors as percentages, the root mean square errors (RMSE) were divided by the range of values of each respective dataset, as follows:

RMSE (%) = (RMSE/(y_max_ − y_min_)) × 100 (2)

The coefficient of determination can also provide a useful indication of the accuracy of the models. As mentioned before, it was determined for each compound at each step of model development: R^2^_C_ for the calibration, R^2^_CV_ for the cross-validated models and R^2^_P_ for the models tested with prediction datasets.

Additionally, the models’ predictive ability was assessed using the range error ratio (RER) and residual predictive deviation (RPD) parameters, as defined in Equations (3) and (4), respectively. These dimensionless parameters are considered indicators of a good model’s ability, as described in the literature [23,24].

RER = (y_max_ − y_min_)/RMSEP (3)

RPD = (Standard Deviation of validation set)/RMSEP(4)

To complete the evaluation of the results obtained, the limit of detection (LOD) was also calculated according to Equation (5):

LOD = 3 × RMSEP(5)

Before the application of PCA and PLS, all datasets were subjected to mean centering. All calculations were performed using Matlab version 8.3 (MathWorks, Natick, MA, USA).

## 4. Conclusions

The early detection of some of the most common off-odors in wines is of paramount importance in the wine industry. The methodology described in this work using FTIR spectroscopy has demonstrated its capability to determine all tested parameters with good accuracy. Indeed, all the PLS models developed yielded R^2^_P_, RER and RPD values higher than 0.9, 10 and 3, respectively, indicating the effectiveness of the developed models. However, only two of the modeled parameters, namely isobutyric acid and acetoin, presented LOD values below the threshold limit in wines. It is important to highlight that these threshold limits were established using wine model solutions. In this context, we believe that assessing the perception of these compounds in wines would be more challenging due to the complexity of the wine matrix. Therefore, the LOD values obtained may be suitable for application in the wine industry, given that this work was performed with a real sample. Moreover, this study serves as a feasibility study, and further research with a wide number and diversity of wine samples is necessary to confirm the robustness of the proposed models. Nevertheless, the results obtained are promising.

## Figures and Tables

**Table 1:** List of the compounds under investigation, responsible for some of the most common off-odors in wine. Chemical and structural formulas. Associated odor description [18,19,20].

Compound	Chemical Formula	Odor Description	Structural Formula
Isoamyl alcohol	C_5_H_12_O	Cheese, burnt alcohol	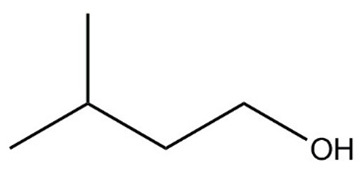
Isobutanol	C_4_H_10_O	Fusel, alcohol, nail polish, oily, bitter, green	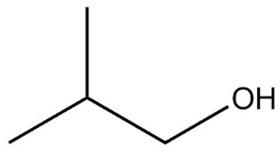
1-hexanol	C_6_H_14_O	Herbaceous, green, grass	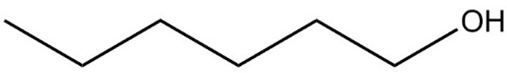
Butyric acid	C_4_H_8_O_2_	Rancid, cheese, sweat	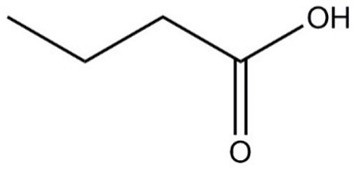
Isobutyric acid	C_4_H_8_O_2_	Fatty, rancid, butter, cheese	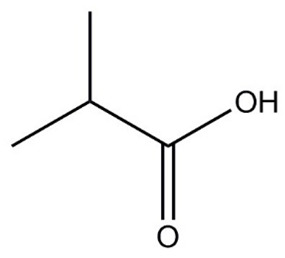
Decanoic acid	C_10_H_20_O_2_	Fatty, rancid	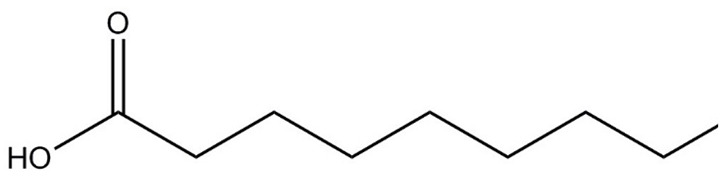
Ethyl acetate	C_4_H_8_O_2_	Fruity, solvent, balsamic	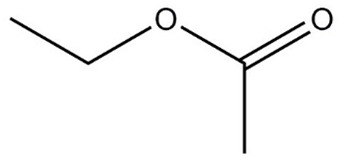
Acetoin	C_4_H_8_O_2_	Buttery cream, flowery, wet	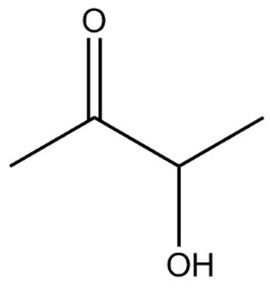
Furfural	C_5_H_4_O_2_	Pungent	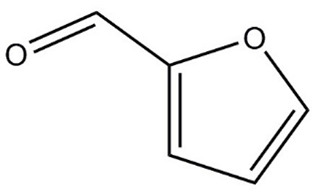

**Table 2:** Summary of the developed PLS models.

	Model		Calibration Set				Prediction Set					
Parameter	LVs	Spectral Region (cm^−1^)	RMSEC (mg/L)	R^2^_C_	RMSECV (mg/L)	R^2^_CV_	RMSEP (mg/L)	RMSEP (%)	R^2^_P_	RER	RPD	LOD (mg/L)
Isoamyl alcohol	3	1580–1201	48.37	0.91	50.57	0.90	40.35	7.9	0.92	12.2	3.3	121.1
Isobutanol	6	3050–2601	37.30	0.95	45.44	0.93	34.05	6.6	0.95	14.7	3.1	102.2
1-hexanol	9	3050–2601	35.76	0.95	46.03	0.91	31.61	6.0	0.96	16.2	3.8	94.8
Butyric acid	7	1580–1201	23.30	0.94	30.95	0.90	28.90	9.8	0.91	10.1	3.6	86.7
Isobutyric acid	6	1580–1201	12.85	0.95	16.69	0.92	12.30	8.1	0.95	12.2	4.4	36.9
Decanoic acid	4	1580–1201	49.60	0.91	52.92	0.90	35.78	7.0	0.94	13.8	3.8	107.4
Ethyl acetate	7	1580–1201	10.03	0.98	14.04	0.95	10.42	4.7	0.98	20.5	7.6	31.3
Acetoin	8	1580–1201	25.58	0.97	37.36	0.93	30.91	6.1	0.96	16.4	4.0	92.7
Furfural	6	1580–1201	15.92	0.95	18.80	0.94	12.11	5.2	0.97	19.0	5.6	36.3

**Table 3:** Description of the samples produced in this work including concentration range, odor threshold and number of produced samples.

Compound	Concentration Range (mg/L)	Odor Threshold (mg/L)	Number of Samples
Isoamyl alcohol	4.08–516.09	30 [17]	223
Isobutanol	5.28–522.42	40 [17]	223
1-hexanol	1.09–524.88	8 [17]	223
Butyric acid	0.92–294.35	10 [17]	223
Isobutyric acid	28.59–179.91	200 [17]	125
Decanoic acid	0.97–511.57	15 [17]	223
Ethyl acetate	0.96–221.53	7.5 [17]	223
Acetoin	22.68–529.33	150 [28]	223
Furfural	1.95–234.71	14.1 [28]	223

## Data Availability

The raw data supporting the conclusions of this article will be made available by the authors on request.

## Supplementary Materials

The following supporting information can be downloaded at: https://www.mdpi.com/article/10.3390/molecules29081882/s1, Table S1: Summary of the developed PLS models using the entire spectral region.
