# Supplementary material for: Application of Fourier-Transform Infrared Spectroscopy for the Assessment of Wine Spoilage Indicators: A Feasibility Study
Source: Molecules. 2024 Apr 20;29(8):1882. doi: 10.3390/molecules29081882 (PMC11054220; doi:10.3390/molecules29081882)
Supplement: Supplementary file 1 [file molecules-29-01882-s001.zip › molecules-2914178-supplementary.pdf]

Table S1: Summary of the developed PLS models using the entire spectral region.

	Calibration set			Prediction set					
Parameter	LVs	RMSEC (mg L^{-1})	RMSECV (mg L^{-1})	RMSEP (mg L^{-1})	RMSEP (%)	R^2_P	RER	RPD	LOD (mg L^{-1})
Isoamyl alcohol	3	60.38	70.47	54.79	12.9	0.88	7.7	2.2	164.4
Isobutanol	6	38.51	63.28	61.41	12.0	0.74	8.4	1.9	184.2
1-hexanol	9	48.80	77.54	99.43	19.1	0.66	5.2	1.0	298.3
Butyric acid	7	38.99	46.41	39.30	13.6	0.98	7.3	2.2	117.9
Isobutyric acid	6	33.37	50.00	53.90	36.5	0.10	2.7	0.9	161.7
Decanoic acid	4	53.48	55.66	61.16	12.2	0.77	8.2	2.1	186.5
Ethyl acetate	7	31.28	43.54	41.57	19.4	0.71	5.1	1.9	124.7
Acetoin	8	46.06	79.11	59.97	12.1	0.80	8.3	2.0	179.9
Furfural	6	30.10	46.20	47.78	20.8	0.57	4.8	1.5	143.3
